# Functional Outcomes Following Ankle Fracture Fixation: A Southeast Asian Series in a Tertiary Care Hospital

**DOI:** 10.7759/cureus.117399

**Published:** 2026-10-03

**Authors:** Gennie Lim Yan Cen, Noman Ali, Don Koh, Ing How Moo, Charles Kon Kam King

**Affiliations:** 1 Orthopaedic Surgery, Changi General Hospital, Singapore, SGP

**Keywords:** ankle fracture, functional outcome, orif, patient education, range of motion

## Abstract

Introduction

Early mobilization following ankle fracture open reduction and internal fixation (ORIF) improves long-term patient functionality. Targeted staged training is mainly focused on minimizing long-term joint stiffness. In this study, we prospectively monitored how ankle range of motion (ROM), active and passive, progresses in the postoperative period.

Methods

This was a prospective, observational study on patients undergoing ORIF of ankle fractures by a single surgeon between 2024 and 2025 at Changi General Hospital, Singapore. We collected ankle ROM measurements, both active and passive, at the 6-week, 8-week, and 12-week postoperative visits. ROM of the ankle was measured by a Jamar E-Z Read goniometer. Analysis of variance (ANOVA) and Bonferroni post-hoc analysis were used to examine statistical differences in ankle ROMs over time.

Results

A total of 28 patients were enrolled. After initial attrition, 23 patients were assessed, and due to a further loss to follow-up of seven patients, 16 completed the study and were analyzed. Serial goniometric measurements demonstrated a statistically significant improvement across 12 weeks in total active range of motion (ROM) from 29.90° ± 8.53° at 6 weeks to 38.17° ± 9.51° at 12 weeks (p= 0.020, partial eta squared = 0.229) and total passive ROM from 38.19° ± 10.48° at 6 weeks to 47.56° ± 10.40° at 12 weeks (p= 0.014, partial eta squared = 0.249). Conversely, active lag--representing the discrepancy between passive and active motion arcs--showed no statistically significant change over time (6 weeks: 8.29° ± 5.59° vs. 12 weeks: 9.39° ± 5.56°; p = 0.384, partial eta squared = 0.062), indicating persistent motor inhibition or weakness relative to joint stiffness resolution.

Conclusion

Our study demonstrates that while operative fixation of ankle fractures yields significant structural recovery and passive joint flexibility over 12 weeks, an active motion lag persists. This plateau in active lag suggests that early postoperative stiffness resolution outpaces neuromuscular recovery, highlighting a clinical rationale for future prospective studies to investigate strategies aimed at optimizing active functional recovery.

## Introduction

Ankle fractures are among the most prevalent lower limb injuries, accounting for approximately 9% to 13% of all skeletal fracture presentations, often resulting from high-energy trauma in young adults or low-energy falls in the elderly [1,2]. The primary surgical goal of open reduction and internal fixation (ORIF) is the anatomical restoration of the ankle mortise to ensure joint stability and prevent post-traumatic arthritis [3,4]. However, the period of postoperative immobilization required for bone healing often leads to deleterious secondary effects, including joint stiffness, muscle atrophy, and a significant reduction in range of motion (ROM) [5-7].

The clinical assessment of recovery has traditionally relied on patient-reported outcome measures (PROMs). While valuable, PROMs like the Olerud-Molander Ankle Score (OMAS) are subjective and may not accurately capture the specific mechanical deficits in joint mobility [8,9]. Objective serial goniometric assessment provides a complementary understanding of the physiological recovery of the ankle joint [10]. Current literature remains divided on the optimal timing for weight-bearing and mobilization, with traditional protocols favoring six weeks of immobilization while contemporary "accelerated" protocols advocate for earlier movement [11,12].

Functional recovery is dependent on active ankle ROM, for which the requirements in the sagittal plane increase progressively from steady-state gait to high-intensity athletic maneuvers. Maintaining a minimum of 10° of dorsiflexion is critical for non-compensatory locomotion, whereas running and dynamic sports demand significantly greater mobility to absorb impact and generate propulsive force. The required active ankle ROM for walking gait is 10°-15° of dorsiflexion and 15°-25° of plantarflexion. Running and sprinting require 15°-25° of dorsiflexion and 25°-40° of plantarflexion, while high-demand sports tasks require at least 25°-35° of dorsiflexion and 40°-50° of plantarflexion [13,14].

This study aims to evaluate serial total active and passive ankle ROM recovery and quantify the trajectory of the active motion lag between 6 and 12 weeks following operative fixation of ankle fractures.

## Materials and methods

The study was approved by the Institutional Review Board (IRB) of SingHealth (approval number: 2020-2643). This was a prospective cohort study conducted at Changi General Hospital, Singapore. Adult patients who underwent open reduction and internal fixation (ORIF) for acute closed (unimalleolar, bimalleolar, and trimalleolar) ankle fractures by a single experienced fellowship-trained foot and ankle surgeon (CK) between July 2024 and May 2025 were consecutively enrolled. All surgical procedures were performed according to standard AO principles (anatomical reduction, stable fixation utilizing compression screws, lateral anatomical plates, and syndesmotic tight ropes when syndesmotic instability was identified intraoperatively). Formal written informed consent was obtained from all participants prior to study inclusion.

Inclusion and exclusion criteria

Patient eligibility was determined based on predefined criteria, as summarized in Table 1.

**Table 1:** Eligibility criteria for patient selection

Inclusion Criteria	Exclusion Criteria
Skeletally mature male and female patients (age ≥ 18 years – 80 years)	Open fractures
Unilateral acute, closed (unimalleolar, bimalleolar, and trimalleolar) ankle fractures requiring surgical fixation	Prior ipsilateral ankle trauma or surgery
General medical comorbidities including diabetes and hypertension	Pre-existing neuromuscular disorders affecting gait
	Fractures managed conservatively
	Bilateral ankle fractures
	Concomitant ipsilateral or contralateral lower-limb injuries/polytrauma
	Pathological fractures or underlying metabolic bone disease

Surgical timing and admission status

Operative fixation was performed at a mean of 4.2±2.1 days following injury, after resolution of acute soft-tissue edema (wrinkle sign positive). All cases were planned, semi-elective surgical procedures performed during index trauma admissions. No emergency revascularizations or open salvage fixations were included.

Sample size and power rationale

Sample size was pragmatically determined by consecutive patient accrual during the 11-month study period. A post-hoc power analysis indicated that the final complete cohort (N=16) achieved >80% power to detect large effect sizes (d≥0.85, partial *n*^2^>0.20) in serial ROM changes at α=0.05.

Postoperative care and outcome assessment

All patients followed a uniform, standardized postoperative rehabilitation protocol under the direct guidance of outpatient licensed physical therapists, supplemented by a prescribed home exercise program. Initial non-weight-bearing active and passive ankle ROM exercises (plantarflexion, dorsiflexion, inversion, and eversion) were initiated at 2 weeks postoperatively following wound inspection and suture removal, prescribed at a frequency of 3 sets of 10 repetitions, 3 times daily. Supervised outpatient physical therapy sessions occurred once or twice weekly for 45 minutes per session to monitor the technique and progress joint mobilization. Non-weight-bearing status was maintained for the first six weeks, after which progressive full weight-bearing was permitted based on clinical and radiographic signs of union. Compliance with home-based exercises was reviewed at each follow-up visit.

Active range of motion (AROM) and passive range of motion (PROM) measurements were recorded in degrees at the 6-, 8-, and 12-week postoperative follow-up visits using a standard Jamar E-Z Read goniometer. Measurements were performed with the patient seated upright with 90° hip flexion and 90° knee flexion to minimize gastrocnemius-soleus tension, with the subtalar joint maintained in a neutral position throughout evaluation. To assess persistent functional muscle inhibition relative to joint stiffness, the Active ROM Lag was calculated for each patient as the numerical difference between total passive ROM and total active ROM (Passive ROM-Active ROM).

Statistical analysis

Data were analyzed using IBM SPSS Statistics for Windows, Version 20.0 (IBM Corp., Armonk, NY, US). Normality of continuous variables was assessed using the Shapiro-Wilk test. Continuous data were expressed as mean± standard deviation (SD). Repeated-measures analysis of variance (ANOVA) was conducted to evaluate changes in active ROM, passive ROM, and active ROM lag across the 3 follow-up time points (6, 8, and 12 weeks). Bonferroni post-hoc adjustments were applied for pairwise comparisons. A p-value of < 0.05 was considered statistically significant.

## Results

Patient demographics

A total of 28 patients met the eligibility criteria and were enrolled in the study. Five patients withdrew prior to the first evaluation due to financial constraints, leaving a total of 23 patients who were assessed. Clinic attendance fluctuated across the study period due to time constraints and work schedule conflicts, with 21 patients evaluated at 6 weeks, 17 at 8 weeks, and 18 at 12 weeks. Ultimately, 16 patients successfully attended all 3 postoperative assessments (6, 8, and 12 weeks) and contributed complete data to the final repeated-measures analysis. The cohort consisted of 15 males (65.2%) and 8 females (34.8%), with a mean age of 48.44 ± 18.17 years (range 22-79 years). The mean BMI at 8 weeks post op was 26.37 ± 3.85 kg/m^2^(range 21.14-35.94 kg/m^2^), with the majority of the patients categorized as either overweight (55.6%) or obese (27.8%). The left ankle was the operative site in 13 cases (56.5%), while the right ankle was involved in 10 cases (43.5%). All patient fractures healed completely with no postoperative complications. Baseline demographics are summarized in Table 2.

**Table 2:** Patient demographics and baseline characteristics *Valid percentages used for BMI categories accounting for missing demographic data in five patients.

Characteristic	Value
Total Patients (N)	23
Age (Years), Mean ± SD	48.44 ± 18.17
Gender, N (%)	23
Male	15 (65.2%)
Female	8 (34.8%)
BMI, Mean ± SD	26.37 ± 3.85
BMI Category, N (%)	18
* Normal	3 (16.7%)*
* Overweight	10 (55.6%)*
* Obese	5 (27.8%)*
Injured Side, N (%)	23
Left	13 (56.5%)
Right	10 (43.5%)

Functional outcomes

Serial goniometric measurements of the ankle active and passive range of motion (sagittal plane: dorsiflexion and plantarflexion) were measured using a standard Jamar E-Z Read goniometer, taken at 6, 8, and 12 weeks postoperatively. Patients were evaluated in a seated position with the knee flexed to 90° to eliminate gastrocnemius tightness. Anatomical landmarks were standardized as follows:

Fulcrum/axis: Placed over the lateral aspect of the lateral malleolus.

Stationary arm: Aligned along the lateral midline of the fibula, referencing the fibular head.

Moving arm: Aligned parallel to the longitudinal axis of the fifth metatarsal.

To minimize inter-observer variability and maintain high measurement reliability, all serial goniometric evaluations were performed by a single investigator.

Normality of the data was confirmed using the Shapiro-Wilk test, which verified a normal distribution across all variables (p>0.05). Furthermore, Mauchly’s test of sphericity indicated that the assumption of sphericity was met for active ROM (p=0.138), passive ROM (p=0.260), and active lag (p=0.259).

A repeated-measures ANOVA determined that there was a statistically significant overall improvement in total active ROM across the 12-week recovery period with a large effect size (F (2,30) = 4.463, p = 0.020, partial eta squared = 0.229). Total active ROM progressed from a mean of 29.90° ± 8.53° at 6 weeks to 38.17° ± 9.51° at 12 weeks. Despite this overall significance, Bonferroni post-hoc analysis did not identify statistically significant differences between specific individual time intervals, though the change from week 6 to week 12 trended toward significance (p= 0.083).

Similarly, total passive ROM demonstrated a statistically significant overall increase over time with a large effect size (*F *(2,30) = 4.986, *p*= 0.014, partial eta squared = 0.249). This metric improved from 38.19° ± 10.48° at week 6 to 47.56° ± 10.40° by week 12. Bonferroni post-hoc testing revealed that this improvement was statistically significant specifically between the week 6 and week 12 time points (*p*= 0.019) (Table 3).

**Table 3:** Serial recovery of ankle range of motion following operative fixation Between 6 and 12 weeks, the minimal clinically important difference (MCID) for total active ROM improved by 8.27° (29.90° to 38.17°) and total passive ROM improved by 9.37° (38.19° to 47.56°). Because both active and passive gains exceed the 5° MCID threshold, these gains represent clinically meaningful functional recovery in addition to reaching statistical significance (p < 0.05). *Determined via repeated-measures ANOVA (sphericity assumed). Normality confirmed via Shapiro-Wilk test (p>0.05). Bold values indicate statistical significance (p<0.05). †Bonferroni post-hoc analysis revealed a statistically significant difference specifically between Week 6 and Week 12 (p=0.019 ).

Parameter	Week 6 (Mean ± SD)	Week 8 (Mean ± SD)	Week 12 (Mean ± SD)	ANOVA p-value*	Partial Eta squared
Total Active ROM (°)	29.90 ± 8.53	33.94 ± 7.13	38.17 ± 9.51	0.02	0.229
Total Passive ROM (°)	38.19 ± 10.48	43.94 ± 9.30	47.56 ± 10.40	0.014†	0.249
Active ROM Lag (°)	8.29 ± 5.59	10.00 ± 7.25	9.39 ± 5.56	0.384	0.062

The active lag-representing the discrepancy between passive and active motion arcs-averaged 8.29° ± 5.59° at six weeks and 9.39° ± 5.56° at 12 weeks. The repeated measures ANOVA revealed no statistically significant change in active lag over the designated follow-up period (*F *(2,30) = 0.988, *p *= 0.384, partial eta squared = 0.062), indicating a persistent motor inhibition or weakness relative to the resolving joint stiffness (Table 3).

## Discussion

The primary objective of this study was to evaluate the postoperative functional recovery of ankle ROM following the operative fixation of ankle fractures. Surgical fixation provides adequate mechanical stability to allow for normal soft-tissue healing and the resolution of capsular stiffness [15,16]. The synthesis of the literature indicates that the early postoperative period is a time of rapid but uneven physiological recovery. Patients following an early weight-bearing (EWB) protocol, often initiated within two weeks of surgery, demonstrate statistically superior ROM at the six-week mark compared to those under traditional immobilization [17]. Most of the fractures heal between the six- and eight-week milestones, and active and passive ROM tend to improve significantly as weight-bearing is fully permitted and staged functional training commences by promoting blood flow and preventing joint adhesions. However, the rate of improvement often declines after this initial window, with additional functional gains slowing down significantly after the first few months [18-20].

Several patient and injury characteristics act as independent predictors of the speed of ROM recovery. Older age (over 40-50 years), high BMI, and comorbidities such as diabetes are independent predictors of slower recovery and persistent stiffness [21]. Furthermore, fracture complexity plays a role; trimalleolar fractures consistently demonstrate the poorest ROM and the greatest gait asymmetries at 6 and 12 weeks compared to simpler fracture patterns. Long-term follow-ups indicate that up to 39% of patients feel they have not made a full recovery even five years post-operation [22,23].

A critical finding in the literature is the persistence of "true" functional deficits despite anatomical healing. This trajectory aligns with expected physiological healing, wherein early mobilization facilitates the remodelling of scar tissue and the gradual restoration of joint compliance [24-26]. This plateau highlights that although the structural joint capsule is regaining normal physiological motion, the neuromuscular unit is failing to fully utilize that available range. This phenomenon is likely attributable to arthrogenic muscle inhibition (AMI), pain-related guarding, or secondary disuse atrophy affecting the local musculature [27]. Consequently, while patients show marked initial progress in ankle ROM between 6 and 12 weeks, achieving pre-injury baseline mobility remains a challenge. These findings emphasize that traditional rehabilitation protocols focusing primarily on passive stretching and early mobilization may be insufficient for complete functional recovery [28,29].

In conclusion, while the data demonstrate a statistically significant improvement in both total active and total passive ROM over the 12-week postoperative period, the active motion lag-the discrepancy between passive flexibility and active motor execution-did not significantly improve, indicating a persistent deficit in neuromuscular function despite structural joint recovery. Recognizing this persistent active lag trajectory provides valuable observational baseline data, establishing a clear clinical rationale for future prospective trials to evaluate targeted rehabilitation protocols.

Limitations

Limitations to this study must be acknowledged when interpreting these results:

Sample size: The cohort with complete serial follow-up across all 3 time points consisted of 16 patients, representing a relatively small sample size. While the study was adequately powered to detect large effect sizes in primary ROM outcomes (partial eta squared > 0.20), smaller functional differences or demographic subgroup analyses remain vulnerable to Type II statistical errors.

Unmeasured confounders: The analysis did not strictly control for external variables that heavily influence functional recovery, such as specific fracture severity (e.g., unimalleolar vs. bimalleolar), individual adherence to physical therapy protocols, or postoperative pain scores.

Clinical implications and future directions

Based on the preliminary findings of this small cohort, the following clinical observations and directions for future research are suggested:

Neuromuscular deficit rationale: The persistence of active motion lag from 6 to 12 weeks suggests that passive flexibility recovers faster than active motor control. While specific rehabilitation protocols were not evaluated in this study, these findings provide a clinical rationale for future research to evaluate whether targeted active resistance exercises can help close this passive-to-active gap.

Tracking active motion lag: Orthopedic clinicians and physical therapists may consider monitoring both active and passive ROM during follow-up to identify subtle active motor deficits that might otherwise go unnoticed if evaluating passive range alone.

Future research: Future prospective studies with larger cohorts and longer follow-up windows (e.g., 6-12 months) are needed to determine when active motion lag fully resolves and whether it correlates with long-term patient-reported outcome measures (such as OMAS).

Larger multicenter cohorts: Subsequent investigations should utilize larger, multicenter sample sizes to control for potential confounders such as fracture classification complexity (e.g., bi- vs. tri-malleolar fractures), patient age, and individual adherence to rehabilitation protocols.

## Conclusions

Following operative fixation of ankle fractures, both total active and total passive range of motion (ROM) improved significantly over a 12-week recovery period. However, active motion lag (the discrepancy between passive and active ROM) showed no statistically significant change over time. This demonstrates that during early recovery, gains in passive joint mobility outpace improvements in active movement. These findings highlight a persistent active motion gap in the intermediate postoperative period, providing a clinical rationale for future prospective studies to investigate strategies aimed at optimizing active functional recovery.
